# Effective Small Molecule Antibacterials from a Novel Anti-Protein Secretion Screen

**DOI:** 10.3390/microorganisms9030592

**Published:** 2021-03-13

**Authors:** Mohamed Belal Hamed, Ewa Burchacka, Liselotte Angus, Arnaud Marchand, Jozefien De Geyter, Maria S. Loos, Jozef Anné, Hugo Klaassen, Patrick Chaltin, Spyridoula Karamanou, Anastassios Economou

**Affiliations:** 1 Laboratory of Molecular Bacteriology, Rega Institute for Medical Research, KU Leuven, 3000 Leuven, Belgium; mohamed.soliman@kuleuven.be (M.B.H.); ewa.burchacka@pwr.edu.pl (E.B.); jozefien.degeyter@kuleuven.be (J.D.G.); maria.loos@kuleuven.be (M.S.L.); jozef.anne@kuleuven.be (J.A.); lily.karamanou@kuleuven.be (S.K.); 2 Molecular Biology Department, National Research Centre, Dokii, Cairo 12622, Egypt; 3 Department of Microbiology and Medicinal Chemistry, Wroclaw University of Science and Technology, 50-370 Wroclaw, Poland; 4 Cistim Leuven vzw, Bioincubator 2, Gaston Geenslaan 2, 3001 Leuven, Belgium; liselotte.angus@gmail.com (L.A.); arnaud.marchand@cistim.be (A.M.); hugo.klaassen@cistim.be (H.K.); patrick.chaltin@kuleuven.be (P.C.); 5 Center for Drug Design and Discovery (CD3), KU Leuven R&D, 3000 Leuven, Belgium

**Keywords:** *E. coli*, alkaline phosphatase, small molecule inhibitors, antibacterials, protein secretion

## Abstract

The increasing problem of bacterial resistance to antibiotics underscores the urgent need for new antibacterials. Protein export pathways are attractive potential targets. The Sec pathway is essential for bacterial viability and includes components that are absent from eukaryotes. Here, we used a new high-throughput in vivo screen based on the secretion and activity of alkaline phosphatase (PhoA), a Sec-dependent secreted enzyme that becomes active in the periplasm. The assay was optimized for a luminescence-based substrate and was used to screen a ~240K small molecule compound library. After hit confirmation and analoging, 14 HTS secretion inhibitors (HSI), belonging to eight structural classes, were identified with IC_50_ < 60 µM. The inhibitors were evaluated as antibacterials against 19 Gram-negative and Gram-positive bacterial species (including those from the WHO’s top pathogens list). Seven of them—HSI#6, 9; HSI#1, 5, 10; and HSI#12, 14—representing three structural families, were bacteriocidal. HSI#6 was the most potent hit against 13 species of both Gram-negative and Gram-positive bacteria with IC_50_ of 0.4 to 8.7 μM. HSI#1, 5, 9 and 10 inhibited the viability of Gram-positive bacteria with IC_50_ ~6.9–77.8 μM. HSI#9, 12, and 14 inhibited the viability of *E. coli* strains with IC_50_ < 65 μM. Moreover, HSI#1, 5 and 10 inhibited the viability of an *E. coli* strain missing TolC to improve permeability with IC_50_ 4 to 14 μM, indicating their inability to penetrate the outer membrane. The antimicrobial activity was not related to the inhibition of the SecA component of the translocase in vitro, and hence, HSI molecules may target new unknown components that directly or indirectly affect protein secretion. The results provided proof of the principle that the new broad HTS approach can yield attractive nanomolar inhibitors that have potential as new starting compounds for optimization to derive potential antibiotics.

## 1. Introduction

Development of modern medical diagnostics and therapeutics, vaccination programs, and improved living standards have led to the control and even elimination of many infectious diseases [1]. Antibiotics have been major contributors to this outcome, and are one of the most important discoveries of the pharmaceutical industry of the 20th century [2]. However, their use is currently limited due to the increasing antibiotic resistance of various bacterial strains and to undesirable side effects [1]. Antimicrobial resistance is responsible for an estimated 25,000 deaths and 1.5 billion € in healthcare costs/year in the European Union [3]. Therefore, there is an urgent need to develop new strategies and methods to prevent epidemics.

Finding new antibiotics against new bacterial target proteins is challenging. Novel antibiotic targets should be: (i) essential for bacterial growth, and (ii) ideally, conserved in bacteria, so that antibiotics can have a broad spectrum, but not present in eukaryotes or, if present, should be sufficiently diverged or inaccessible. A parallel approach for new anti-infectives are drugs that inhibit bacterial virulence and/or pathogenesis but that are not essential for viability. Such inhibitors can be part of combination therapies and can boost effective immune responses in the host [4].

**Figure 1:**
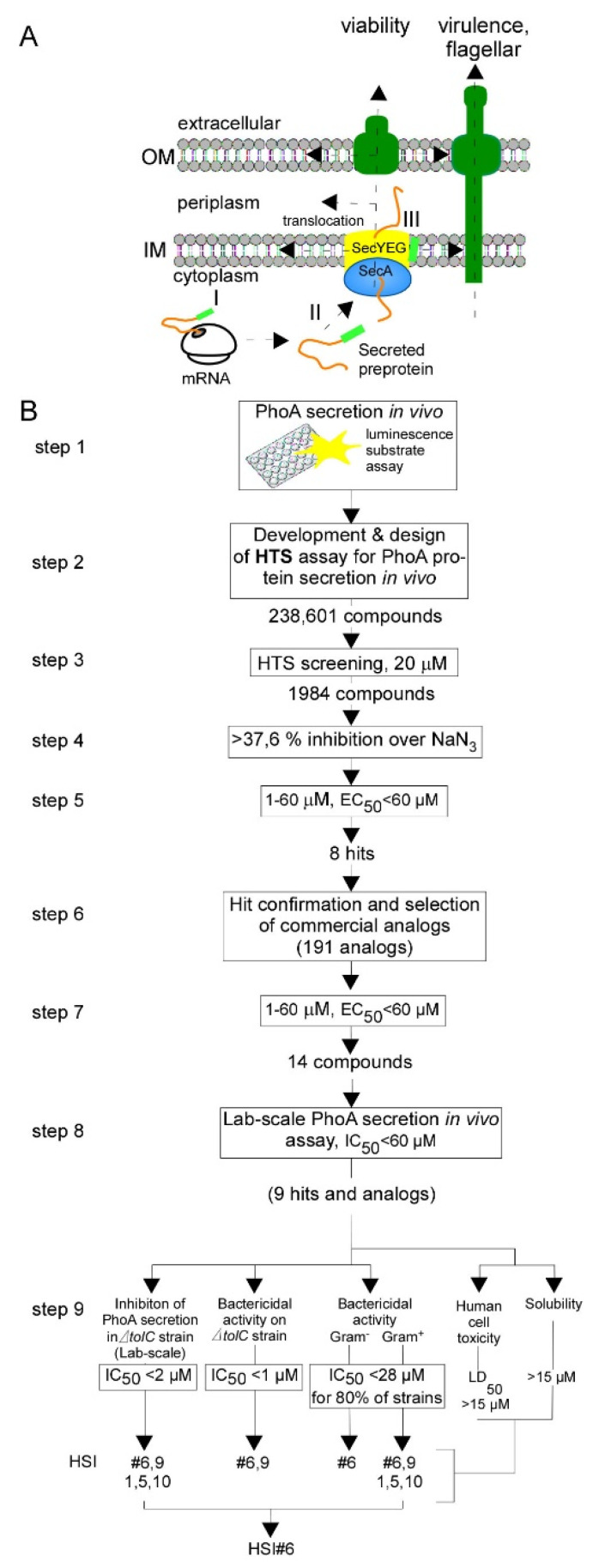
Overview of Sec pathway and HTS pipeline to discover anti-protein secretion. (**A**). Cartoon of the Sec pathway in a cell. (**B**). HTS and screening pipeline used for the identification and characterization of secretion inhibitors.

One important process for both bacterial viability and virulence is protein secretion into and across the plasma membrane using the Sec system (Figure 1A) [5,6]. It mediates the export of 30 to 35% of the bacterial proteome. Sec secretion pathway components such as the ATPase SecA [7], signal peptidases [7], the lipoprotein trafficking system LOL [8], and the BAM outer membrane assembly complex [9] meet wholly or partly the criteria of attractive targets. Many of these proteins have important advantages as drug targets: they are stable in vitro, have rather well-known structure/function features and probably good accessibility due to their location in the cell envelope and membranes [7]. These export machineries are then used by 59 client proteins that are essential for viability that are exported in K-12 MG1655 *E. coli* and are located in the inner membrane and periplasm [10]. Several specialized export machineries, like the Type III secretion system (T3SS) [11,12,13,14], export pathogenicity proteins to the bacterial cell surfaces and beyond [15] and undergo Sec-dependent assembly [16,17].

High throughput screening has been used to identify protein secretion inhibitors as antibacterials or to reduce bacterial virulence [18,19]. These assays were designed to follow the export of reporter enzymes or fluorescent proteins for example: β-galactosidase behind a LamB signal peptide [18]. However, these assays commonly required prolonged incubation times [19] or diplayed high false-positive rates due to colored compounds that interfere with the enzymatic reaction [18].

Similarly, using other assays, different screens were developed such as structure-based virtual ligand; several inhibitors for protein secretion particularly that target SecA have been discovered [7,20,21,22,23,24,25]. Sodium azide, the first known SecA inhibitor [26] is not a usable antibacterial because it is non-selective and inhibits eukaryotic enzymes such as other ATPases [27]. Equisetin (CJ-21,058), 5-amino-thiazolo(4,5-d)pyrimidine, n-(3-(benzyloxy)-5-ethoxybenzyl)-1-(piperidin-4-yl) methanamine (P87-A4) and its analog 2-((3-(benzyloxy)-5-ethoxybenzyl)amino)ethane-1-ol (17D9) inhibit the translocation ATPase (IC_50_ of 23.9-135 µM) [20,21,25].

Other attractive Sec pathway targets include the essential Type I signal peptidase (SPase I) which releases mature secreted proteins from the membrane-embedded SecY channel (Figure 1A, yellow) and has a unique catalytic mechanism absent from eukaryotic serine proteases [7,28,29,30,31].

As terminal Sec pathway branches, the lipoprotein (Lol) and outer-membrane β-barrel proteins (OMPs) are also targets for inhibitors. Some of the inhibitors of the Lol pathway inhibit the chaperone LolA by preventing its binding to substrates [32]. These inhibitors affect *E. coli* strains with a MIC of 16 µg/mL [33,34]. Moreover, S-(4-chlorobenzyl)isothiourea and S-(3,4-dichlorobenzyl)isothiourea (A22) were effective against *E. coli* MG1655 LolA with IC_50_ of 150 and 200 µM, respectively [35]. For OMPs, β-hairpin macrocyclic peptidomimetic JB-95 was described as an inhibitor for the BamA insertase and affects *E. coli* ATCC25922 with a MIC of 0.25 µg/mL [36].

Here, we took a more general non-targeted approach aiming at inhibitors against the whole process of Sec-dependent post-translational protein secretion using *E. coli* as a screening model bacterium in vivo. To identify unknown new targets, we followed the periplasmic secretion of alkaline phosphatase (PhoA). PhoA is only active in this sub-cellular location after dimerization, and disulfide oxidation [37,38,39,40], and therefore, becoming active necessitates efficient secretion. To monitor its activity, we developed a high-throughput screening (HTS) luminescence-based assay and used it against a library of ~240K small molecules. After hit confirmation and selection of analogs, fourteen compounds (HSI; HTS secretion inhibitors) belonging to eight different chemical series and displaying an IC_50_ ranging from 3 to 60 µM were determined in the secretion of alkaline phosphatase assay. The compounds were also tested as inhibitors of viability of either Gram-negatives or positives, including of 16 bacterial species (Gram-positive and Gram-negative) from the WHO’s top pathogens list [41].

Seven of the inhibitor compounds identified in the HTS had microbicidal activity and represented three structural families HSI#9(parent) and 6, HSI#1(parent), 5, and 10, and HSI#12 and 14. HSI#6 of the first structural family inhibited the growth of eight Gram-negative and five Gram-positive bacteria with excellent micromolar IC_50_ values of 0.4 to 9 µM, while HSI#9 showed inhibition in the growth of four Gram-positive strains and *E. coli* strains with IC_50_ < 27 µM. HSI#1, 5, and 10 of the second structural family revealed antibacterial activity toward Gram-positive (IC_50_ of ~7–38 μM) and of *E. coli* strain BW25113Δ*tolC* (IC_50_ < 14 μM), which suggested that outer membranes hampered their permeability. HSI#12 and 14 of the third structural family inhibited only the growth of *E. coli* strains (IC_50_ of 44-65 μM). Under our assay conditions, none of the tested compounds inhibited the SecA ATPase activities in vitro; therefore, the observed inhibition is not SecA-specific but rather targets additional unknown components that affect the secretion process.

In summary, our in vivo screening approach using an *E. coli* lab strain returned a wide range of promising anti-bacterials with broad and narrow spectrum properties.

## 2. Materials and Methods

### 2.1. Small Compound Library

The library was provided by HDC and contains around 240,000 drug-like and lead-like compounds, carefully selected by a team of medicinal and computational chemists to provide the best chemical starting points for drug discovery. The collection consists of three subsets (discovery set, explorer set, and probe set) which, taken together, provide an excellent diversity for drug discovery projects. More information can be found here: https://www.hit-discovery.com/services/ (accessed on 15 December 2020).

### 2.2. HTS Assay for Bacterial PhoA Secretion In Vivo

The *E. coli* strain BL21 (pLysS) was transformed with pET22b plasmid [42] (pIMBB882) carrying PhoA and grown overnight in 5 mL LB containing Ampicillin (100 µg/mL) and Chloramphenicol (25 µg/mL) by taking a stab of the frozen glycerol stock and suspending it in the LB medium at 37 °C. This was shaken at 250 rpm. The next day, 5 mL of the pre-culture was transferred into 200 mL LB medium and grown for another 2.5 h at identical conditions until the OD_600_ reached 0.6. 25 µL of this cell suspension was dispensed into full white 384 well assay-ready plates containing 300 nl from 2 mM stock of the compounds (final concentration 20 µM). The 384 well plates were shaken vigorously for 30 s and incubated at 30 °C for 15 min. After this pre-incubation, the PhoA expression was induced by the addition of 5 µL (0.1 mM) IPTG followed by incubation (1.5 h; 30 °C). Next, 5 µL of CellLytic express (Sigma-Aldrich, St. Louis, MO, USA) was added, shaken for 30 s, and incubated at 20 °C for 15 min. PhoA was detected by the addition of 25 µL of AP-juice that contains 1,2-dioxetane-based chemiluminescent enzyme substrates [43] (p.j.k. GmbH, Kleinblittersdorf, Germany), shaken for 60 sec and incubated (10 to 20 min), followed by luminescence measurement on an Envision luminometer (Perkin Elmer, Waltham, MA, USA). AP-juice allows 1, 2 dioxetane to produce light upon its degradation with alkaline phosphatase [44,45].

### 2.3. HTS Screening

All compounds were dissolved in DMSO (20 mM stock) and tested at a final concentration of 20 µM in 384 well plates containing 320 compounds, 32 negative controls (DMSO alone) and 32 positive controls (Sodium Azide 4 mM). Controls were used to calculate for every compound the percentage of inhibition relative to the controls, as well controls used to define the quality of the experiment per plate by calculation of the Z’-score and signal-over-background ratio (S/B). All plates screened had a Z’-score higher than 0.5 with an average of 0.78, and an average S/B of 25.8.

To exclude molecules that are giving false-positive results, we developed a counter screen that was based on the same detection methodology as the primary PhoA screening assay. For this, 300 nL of compound/vehicle per well was spotted into a white 384 well plate with a solution of Alkaline Phosphatase (EF0651, Thermo Scientific, Waltham, MA, USA) in PBS (with Ca^2+^ and Mg^2+^) dispensed (30 µL) and shaken. After 1 to 5 min incubation, 25 µL of AP-juice was added to the samples followed by a shaking step for 1 min and finally read on an Envision. Any compound that inhibited the PhoA activity in a dose-dependent manner was excluded from further evaluation.

### 2.4. Solubility Assay

The determination of the aqueous solubility of compounds in this assay is based on the principle of turbidimetry. Turbidimetric methods rely on the measurement of light scattering from precipitate in solution to determine the solubility. Precipitation is identified by an absorbance increase due to blockage of the light by the particles at the wavelength of 570 nm. Compounds, stored in matrix vials (Thermo Fisher) at a stock concentration of 30 mM or 10 mM in 100% Dimethyl sulfoxide (DMSO), were used to make a serial dilution (dose-response) in a 96-well v-bottom propylene plate (Greiner, 651201). Serial dilutions are made to perform the solubility assay; they were made row-wise and start with undiluted compound (30 mM or 10 mM) in the first well and were then 1 over 3 diluted further on. The serial dilutions were eight doses long and contain six compounds per plate maximal (rows B-G). Columns 1 and 12 were filled with 100% DMSO for control purposes. The dose-response plates were 200-times diluted in 300 µL Phosphate Buffered Saline (PBS; pH = 7–7.2 without Ca^2+^/Mg^2+^) (Gibco, 14190-144) by transferring 1.5 µL of serially diluted compound. This results in final starting concentration of 150 µM (starting from 30 mM) or 50 µM (starting from 10 mM) at 0.5% DMSO. Dilutions were made by diluting one 96-well plate in two 96-well, flat-bottom, polystyrene plates (Greiner, 655101). These plates were then incubated at room temperature for 1h. After 1h incubation, the plates were read on the envision (Perkin Elmer) at 570 nm.

The data of this assay is reported as “Soluble at” value for each compound. This “soluble at” value represents the concentration where the compound is still soluble and is the concentration before the first precipitated concentration. The first precipitated concentration is the concentration where the absorbance value is more than five standard deviations higher than the average background absorbance. The average background absorbance and the standard deviation are calculated on the 0.5% DMSO controls in columns 1 and 12.

### 2.5. Cytotoxicity Assay

Hek293T cells were harvested and diluted obtaining a cell suspension with a concentration of 200,000 cells/mL in complete growth medium (DMEM, 4.5 g/L d-glucose, pyruvate 1 mM, 0.075% bicarbonate and 10% Fetal Bovine serum). Of this cell suspension, 50 µL (10,000/well) was seeded in a 384 well culture plate (white polystyrene, tissue culture treated) and incubated at 37 °C-5% CO_2_ for 4 h in a moisturized incubator, which enables the cells to adhere. After the incubation, small chemical compounds were diluted in medium and 10 µL of a compound solution was added onto the cells, ending up with the required compound concentration. The cells were incubated for 24 h at 37 °C-5% CO_2_ in a moisturized incubator where after × µL medium was removed to equalize the liquid levels within one plate ending up with ±25 µL/well. The amount of remaining cells/well was determined with ATPlite^TM^ 1step (Perkin Elmer) Adenosine TriPhosphate (ATP) monitoring system, which is based on firefly luciferase.

The cytotoxicity was calculated by subtracting RLU (relative light units) obtained from the cells incubated with a compound from the RLUs obtained from cells in the presence of the vehicle.

The cytotoxic effect of a test compound was determined as:

Percent cell death = [1−((RLU determined for sample with test compound present—1) divided by (RLU determined in the presence of vehicle—1))] * 100.

### 2.6. Antimicrobial Activity Test

The antibacterial activity of compounds against various bacterial strains (*S. aureus* ATC6538P, *B. subtilis* ATCC6633, *E. coli* BL21, *E. coli* MC4100, *E. coli* BW25113, *E. coli* BW25113Δ*tolC*, *E. coli* BW25113Δ*lptD* and Entheropathogenic *E. coli O127:H6 (strain E2348/69/EPEC)*) was measured using the serial dilution method in microplates. An overnight culture of all tested bacteria in LB medium was diluted 200-fold in fresh LB medium and incubated at 37 °C until the OD_600_ reached 0.3. Nine strains of the WHO’s top 16 pathogens list, *Pseudomonas aeruginosa* 3/88, *Klebsiella pneumoniae* ATCC 27799, *Enterobacter cloacae*, *Proteus vulgaris*, *Providencia stuartii*, *Morganella morganii*, *Serratia marcescens*, *Salmonella typhimurium* and *Shigella sonnei* were grown in LB medium, while *Mycobacterium abscessus* ATCC19977, *Enterococcus faecium* ATCC 804B, *Campylobacter jejuni*, *Streptococcus pneumoniae*, *Haemophilus influenzae* and *Mycobacterium abscessus* were grown in tryptic soya broth (TSB) and incubated at 37 °C and 5% CO_2_ until the OD_600_ reached 0.3 as previously discussed. 

Next, 20 μL of this culture, which was previously diluted to OD_600_ < 0.01, was added to a 96-well microtiter plate containing different concentrations of each compound in the range of 0 to 100 μM (final DMSO concentration 2.5% (v/v); final volume of 200 µL) or DMSO alone (2.5% (v/v)). Bacterial cultures were incubated at 37 °C for 20 h with no shaking, OD_600_ was measured spectrophotometrically (Tecan Infinite^®^ 200 PRO) and the data were normalized against a control culture [0 μM compound, 2.5% (v/v) DMSO]. IC_50_ values were calculated in GraphPad Prism by nonlinear regression using equation model: Y = Y_Bottom_ + (Y_Top_ − Y_Bottom_)/(1 + 10^((Log IC_50_ − X)*(−1.0))^ where Y_Bottom_ and Y_Top_ are plateaus in the units of the Y axis. The IC_50_ gives a response halfway between Y_Bottom_ and Y_Top_ and thus measures the potency of a compound in inhibiting bacterial viability and indicates reduction of bacterial growth by 50%.

## 3. Results

### 3.1. Development of an In Vivo HTS Assay for E. coli Protein Secretion

For the HTS, we used *E. coli* strain BL21 expressing and secreting alkaline phosphatase (PhoA). Measurement of PhoA activity was used to monitor post-translational protein secretion. PhoA becomes enzymatically active only once translocated to the periplasm via a functional Sec machinery. In the presence of Sec pathway inhibitors, PhoA would not be translocated and phosphatase activity should be reduced. We developed a sensitive 384 well setup phosphatase assay amenable to high throughput screening using “AP-juice” (P.j.K GmbH) as a phosphatase substrate that can be monitored in a luminometer once hydrolyzed (Figure 1B) (see Materials and Methods). The PhoA gene was expressed behind IPTG-inducible T7 RNA polymerase control on plasmid pIMBB882. The amounts of IPTG and the number of cells used were optimized for the highest signal-to-noise ratio at 30 °C. As a positive control (i.e., maximal inhibition), we used sodium azide [46]. Cells treated with the DMSO vehicle alone served as a negative control.

### 3.2. HTS Results

To identify inhibitors that affect PhoA secretion, we tested a small molecule library of 238,601 compounds using the in vivo PhoA assay. The compounds were dissolved in 100% DMSO at 20 to 30 mM and tested at a final concentration of 20 µM [47]. Under our HTS assay conditions, sodium azide inhibited the secreted phosphatase activity by >90%. As an additional control, we used CC#02 (quinazoline-derivative) that was previously selected in an HTS assay based on β-galactosidase export across the inner membrane [48]. CC#02 inhibited periplasmic export of β-galactosidase by 50% and β-lactamase by 34% but did not directly affect SecA and had little antibacterial activity [48].

**Figure 2:**
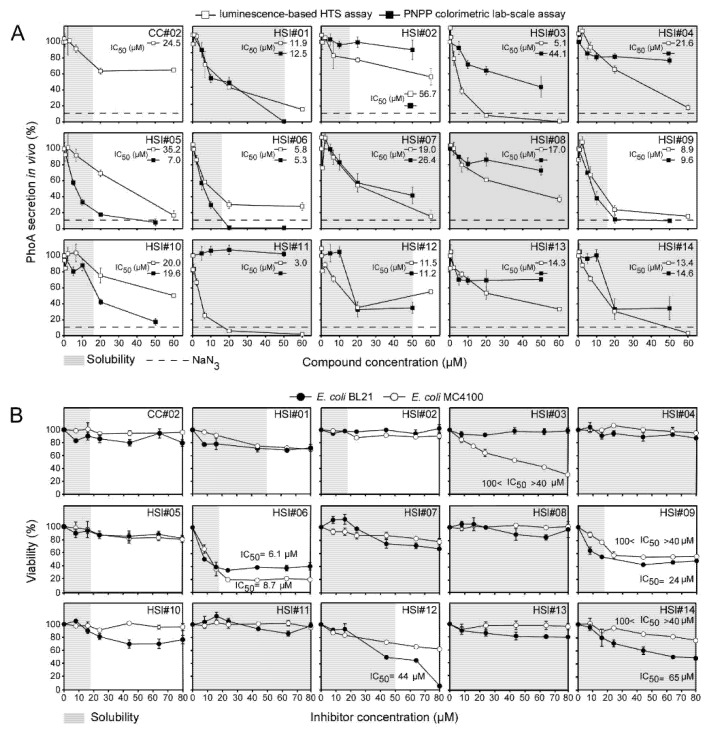
In vivo anti-PhoA secretion and antibacterial activity of secretion inhibitors toward *E. coli* strains. (**A**). The effect of the 14 compounds discovered by HTS and CC#02 at different concentrations on PhoA secretion in vivo tested using the luminescence (HTS) and the *p*-nitrophenyl (lab-based) PhoA actvity assays. PhoA secretion in the absence of any compound but in the presence of DMSO 2.5% (v/v) was set as 100%. (**B**). Inhibition of bacterial viability by the indicated inhibitors and CC#02. The growth of the indicated *E. coli* strains was measured at OD_600_ (OD_600_ in the absence of any compound but in the presence of 2.5% (v/v) dimethyl sulfoxide was taken as 100% and OD_600_ in the presence of inhibitor was normalized to it) was plotted against the inhibitor concentration. IC_50_ values for growth inhibition are indicated. *n* = 3. The results are presented as the mean ± SD. Gray shade: aqueous solubility.

A total of 1984 compounds out of the entire library inhibited the PhoA activity by > 37.6% relative to the positive (sodium azide) and negative control (DMSO). These were next tested in a dose-dependent manner up to 60 µM. The dose-response testing yielded eight molecules representing eight structural families that showed dose-dependent inhibition of in vivo PhoA secretion. A total of 191 analogs of these eight hits (resupplied for independent confirmation) were selected and tested in the in vivo PhoA secretion assay leading to the identification of six more compounds, a total of 14, representing eight families with dose-dependent inhibition (Figure 2A). None of these inhibited the enzymatic activity of purified native phosphatase in a counter assay (not shown). Moreover, proPhoA was detectable by immunoblotting in cells treated with the strongest inhibitors (Figure S1); therefore, the effects of the compounds must lie downstream of proPhoA synthesis.

The 14 HSI compounds of interest were re-purchased and their inhibitory activity re-confirmed in the luminescence-based in vivo secretion assay. All inhibited PhoA secretion (IC_50_ < 57 µM; Figure 2A; Table 1). Three of them vary significantly (HSI#3, 6 and 11; IC_50_ of 3–5.8 µM; Figure 2A; Table 1), nine of them and CC#02 significantly (IC_50_ of 8.9–24.5 µM) and two weakly (HSI#5 and 2; IC_50_ of 32 and 56.7 µM, respectively). Ten of the compounds displayed very similar (HSI#1, 5, 6, 9 and 10) or similar (HSI#3, 4, 7, 12 and 14) trends in a lab-scale in vivo PhoA secretion assay [49,50](see Supplementary Materials and Methods) monitoring *p*-nitrophenyl phosphate production (Figure 2A, filled squares; Table 1), while the remaining four compounds showed marginal effects.

Next, the HSI compounds were examined for solubility and cytotoxicity and microbicidal activity against several species and strains.

### 3.3. Solubility and Cytotoxicity Testing of the HSI Compounds

The kinetic aqueous solubility of the 14 compounds was determined using a turbidimetric method, measuring an increase in the absorbance (at 570 nm) of scattered light resulting from compound precipitation. CC#02, HSI#5 and 10 displayed low solubility, (4 to 5 µg/mL; ~16.7 µM) (Table 1), close to the minimal solubility recommended for drugs (U.S. Pharmacopeia, [51,52]. HSI#2, 6, and 9 showed solubility just over 5 µg/mL (16.7 µM) while the remaining nine compounds showed higher solubility (10 to 60 µg/mL; 50 to 150 µM) (Table 1).

The cytotoxicity of the compounds was tested toward HEK293T cells by determining the number of remaining live cells with an ATP monitoring system (see Materials and Methods). HSI#1, 5, and 10 showed the highest toxicity levels (LD_50_ = 6.7 µM); this likely compromises their use for further development (Table 1).

### 3.4. Effect of HSI Compounds on the Viability of E. coli Strains

We next determined the in vivo antibacterial properties against Gram-negative bacteria for the 14 compounds and the controls using small-volume growth in 96-well plates (Figure 2B).

Sodium azide inhibited growth by ~80% at 4 mM, while CC#02 barely inhibited the growth of any of the *E. coli* strains (Figure 2B; Table 1). In the absence of any other relevant indicator of secretion inhibition, sodium azide sets an empirical boundary of what level of anticipated secretion inhibition might be lethal for ~80% of the cells. At the concentrations used, sodium azide might have pleiotropic effects.

The viability of *E. coli* strains BW25113 (see below), BL21 and MC4100 was inhibited significantly by HSI#6 (IC_50_ of 6.1–8.7 µM; Figure 2B; Table 1) and more weakly from HSI#9, 12 and 14 (24 to 100 µM; Figure 2B; Table 1). HSI#6 was equally active in the 96-well plate growth assay and on LB agar plates (Figure S1b). On the contrary, none of the 14 compounds affected the growth of Enteropathogenic *E. coli* (Figure S2).

### 3.5. Effect of the Compounds on the Viability of Gram-Positive Bacteria and the WHO Top Critical Pathogens

**Figure 3:**
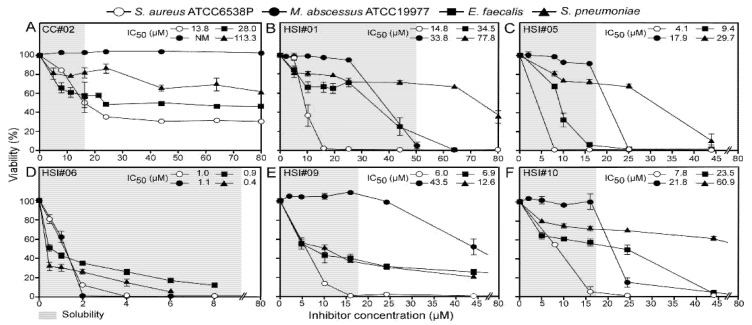
Antibacterial activity of the secretion inhibitors toward Gram-positive bacteria. (**A**–**F**). Inhibition of bacterial viability by the indicated inhibitors and CC#02. Growth of the indicated Gram-positive strains was plotted against the inhibitor concentration (as in Figure 2B). IC_50_ values are indicated. *n* = 3. The results are presented as the mean ± SD. Gray shade: aqueous solubility.

We next tested the compounds on the viability of Gram-positive bacteria: a non-pathogenic *B. subtilis* ATCC6633 lab strain and four strains closely related to ones from the WHO’s top 16 pathogens list: *S. aureus* ATCC 6538P, *Enterococcus faecalis* ATCC 804B, *Streptococcus pneumoniae* and *Mycobacterium abscessus* ATCC19977 (Table 1 and Table 2). Sodium azide barely inhibited the growth of most Gram-positive bacteria (any observable inhibition commonly required >100 mM; Table 1). CC#02 inhibited growth of three Gram-positive bacteria well (IC_50_ of 13 to 28 µM) but not of *M. abscessus* (Figure 3A). As with Gram-negative bacteria, HSI#6 showed the highest antibacterial effect toward Gram-positive bacteria (IC_50_ of 0.4 to 2.4 µM) (Figure 3D; Table 1 and Table 2), with *S. pneumoniae* being two to three times more sensitive. Moreover, HSI#1, 5, 9, and 10, displaying limited effects against Gram-negative bacteria, showed high antibacterial activity toward *B. subtilis, S. aureus*, *M. abscessus* and *E. faecalis* (IC_50_ of ~4 to 38 µM) (Figure 3B,C,E,F; Figure S3). The IC_50_ values of HSI#1 and 10 were, respectively, higher for *S. pneumoniae* compared to those for other Gram-positive bacteria.

**Figure 4:**
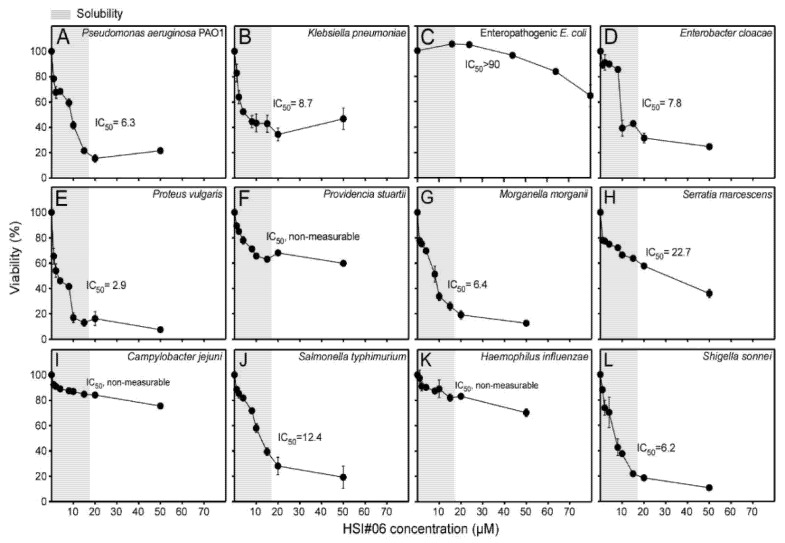
Antibacterial activity of secretion inhibitors toward 12 Gram-negative bacteria from the WHO’s top 16 pathogens list. (**A**–**L**). The indicated inhibitors which revealed inhibition of Gram-negative bacterial viability. The growth of the indicated bacterial strains (as in Figure 2B) was plotted against the inhibitor concentration. IC_50_ values for growth inhibition of the bacteria are indicated. Growth in the presence of 2.5% (*v/v*) dimethyl sulfoxide in the absence of inhibitors was taken as 100%. *n* = 3. The results are presented as the mean ± SD. Gray shade: aqueous solubility.

HSI#6 inhibited the viability of *E. coli* and the five Gram-positive bacteria, and also inhibited the viability of eight of the 12 Gram-negative bacterial strains of the WHO top 16 list [41] (Figure 4; Table 2) with IC_50_ values of ~3 to 22 µM (Figure 4A–D,F,G,J,K; Table 2).

### 3.6. Effect of HSI Compounds on the Viability and Secretion of E. coli Strains with Compromised outer Membranes

The outer-membranes of Gram-negative bacteria are a significant obstacle to novel antibacterial compound discovery [53]. As HSI#1, 5 and 10 inhibit only Gram-positive growth (Figure 4), we aimed to determine whether either these molecules are selective Gram-positive antibacterial or if the Gram-negative outer membrane prevents their permeability. In that context, the *E. coli* strains with compromised outer membranes BW25113*::imp-2413^+^* [54] and BW25113Δ*tolC* [55], which show increased permeability [53], were used.

**Figure 5:**
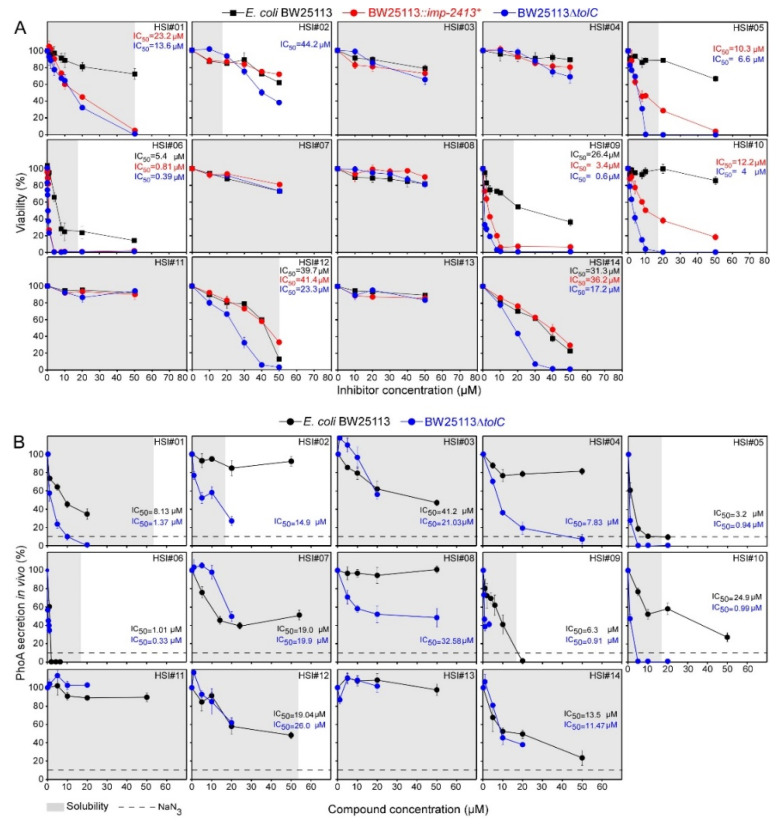
Antibacterial activity and in vivo PhoA secretion inhibition toward *E. coli* strains and derivatives. (**A**). Antibacterial activity toward *E. coli* BW25113 and its derivatives BW25113Δ*tolC* and BW25113*::imp-2413^+^* of the indicated 14 inhibitors isolated from the HTS screening. *n* = 3. Results are presented as the mean ± SEM. Gray shade: aqueous solubility. (**B**). The effect of the 14 compounds isolated from the HTS screening on PhoA secretion in vivo of the indicated strains tested using the p-nitrophenyl assay (as in Figure 2A). The SecA inhibitor sodium azide (4 mM) [26] was used as a positive inhibitory control for maximal SecA inhibition observable in vivo (dashed line). Gray shade: aqueous solubility.

The BW25113 derivatives Δ*tolC* and *imp-2413^+^* showed higher sensitivity toward seven of the compounds (HSI#1, 5, 6, 9, 10, 12, and 14) by 2 to 40 times compared to BW25113, with Δ*tolC* cells showing in most cases stronger susceptibility (Figure 5A).

Outer-membrane crossing reduced maximal inhibitory effect with nine compounds inhibiting PhoA secretion in BW25113Δ*tolC* with IC_50_ lower than that determined in BW25113 (HSI#1-6 and 8–10; Figure 5B; Table 1). HSI#1, 5, 6, 9, and 10 inhibited PhoA secretion in BW25113Δ*tolC* with IC_50_ lower than that of the WT by 90%. HSI#2, 4, and 8 inhibited PhoA secretion only in BW25113Δ*tolC*.

Apparently, for some compounds, outer membrane permeability was an obstacle in reaching sufficient concentrations in the cell to be inhibitory.

### 3.7. Effect of Compounds on SecA ATPase In Vitro

To determine if the effect of the 14 compounds might be exterted directly on SecA, we measured their ability to inhibit the SecA-ATPase activity in vitro. However, none of the compounds directly inhibited SecA ATPase activities in vitro (Figures S5 and S6), and thus, the inhibition of PhoA secretion seen resulted from an effect on a different target.

### 3.8. Chemical Characterization of Derived PhoA Secretion Inhibitors

**Figure 6:**
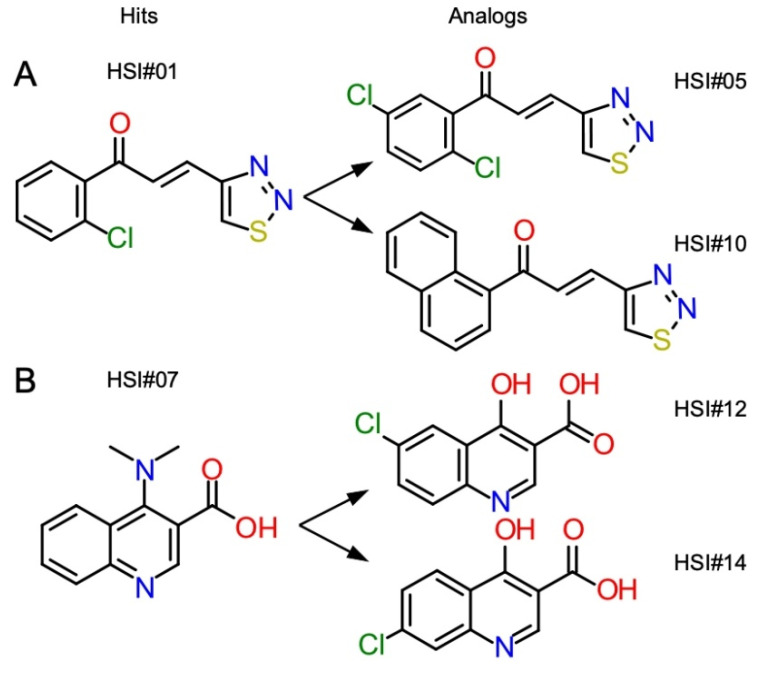
Compound structures of HSI#1, 5, and 10 (**A**) and HSI #7, 12 and 14 (**B**).

HSI#1(parent), 5, and 10 belong to the same chemical series (Figure 6A). The three active analogs bear a 1,2,3-thiadiazole ring connected to a lipophoilic aromatic moiety with an acrylate linker, making them potential Michael acceptors. Early SAR data gathered from the commercial tested analogs showed that this acrylate linker seemed to be essential for activity (data not shown) but more work will have to be done to confirm that initial observation.

HSI#7 (parent), 12, and 14 belong to the same chemical series (Figure 6B), the three active analogs are derivatives of quinoline-3-carboxylic acid. Different derivatives of 3-quinolinecarboxylic acid have been reported to exhibit antimalarial [56,57] and antibacterial activities against both Gram-negative and Gram-positive [58,59].

HSI#6 and 9 will be characterized in-depth in a future study.

## 4. Discussion

We present a multi-step pipeline to identify novel inhibitors that display strong antibacterial activity and were identified in an anti-protein secretion screen (Figure 1B). This pipeline returned 14 compounds from eight structural families that inhibited PhoA secretion with IC_50_ < 50 µM (Figure 2A), and seven of which showed strong antibacterial activity. Therefore, broad-spectrum nanomolar antibacterials can be identified in such broad in vivo assays that screen anti-protein secretion.

Five compounds (HSI# 1, 5, 6, 9, and 10) detected with anti-protein secretion assay (IC_50_ of ~5–35 µM) were effective antibacterials (IC_50_ of ~1–37 µM) with HSI#6 being the most effective inhibitor toward both Gram-positive and Gram-negative bacteria. IC_50_ values are comparable with those of commercially available antibiotics, i.e., vancomycin, penicillin G, and ampicillin (IC_50_ = 17.0, 0.5, 0.01 µM toward *S. aureus*, respectively; Figure S4). Given its only moderate toxicity on human cells (Table 1) and relatively broad-spectrum, HSI#6 may be a good lead for further optimization.

Three of the five compounds above (HSI#1, 5 and 10) displayed significant activity against Gram-positive bacteria (Figure 3A). This revealed that although a Gram-negative bacterial model strain was used for screening, our approach can return potent Gram-positive antibacterials. The screening assay is sensitive enough to pick out multiple, broad antibacterial compounds. These compounds reduced PhoA secretion in *E. coli* (Figure 2A) but did not affect viability (Figure 2B) suggesting inefficient penetration through the Gram-negative outer membrane. This is common for many antibiotics that are highly effective against Gram-positive bacteria (e.g., macrolides, novobiocin, rifamycin, lincomycin, clindamycin and fusidic acid; [60]). Strains missing outer membrane proteins [61] increased the susceptibility of *E. coli* viability for eight compounds including the three antibacterials originally only active in Gram-positive bacteria (HSI#1, 5, and 10) and to the previously non-effective compound HSI#2 (Figure 5A). Additionally, the *tolC* knock-out enhanced the potency of nine compounds as PhoA secretion inhibitors in vivo (Figure 5B). This suggested that using the *tolC* mutant strain could be useds as a tool for the selection of broad antibacterials. As HSI#1, 5, and 10 display high-level HEK293T toxicity, they are not attractive for further optimization.

The remaining nine compounds were moderate to strong PhoA secretion inhibitors but only two of them (HSI#12 and 14) affected the growth of *E. coli* marginally with an IC_50_ > 45 µM. As protein secretion is an essential process, we consider two possibilities for these false positives. Some HSI compounds might affect the folding and/or formation of disulfides of periplasmic PhoA polypeptides that have already been secreted or are being secreted; enzymes such as the Dsb proteins are known catalysts of disulfide oxidation but are not essential for viability [10,62]. They might also compromise the secretion process directly but not sufficiently so as to yield a substantial antibacterial effect. It should be noted that even sodium azide, a potent *E. coli* anti-bacterial at 3 to 4.6 mM [47], still yields a substantial level of secreted PhoA (~17%; Figure 2A). Therefore, unless secretion is inhibited at such levels, it will not lead to lethality. In most cases, the best correlation with sensitivity to a drug was their ability to be taken up at sufficient final intra-cytoplasmic concentrations [63]. For some compounds, maximal usable amounts are limited by their solubility (Table 1). For many essential cellular targets, even a reduction in production by 97% does not lead to lethality [64].

HSI#7, 12, and 14 are quinoline-3-carboxylic acid derivatives (Figure 6B) and might inhibit DNA gyrase A [65,66] as do analogous compounds [58]. Whether and how these activities connect directly or indirectly to protein secretion is unclear. However, such compounds are known to affect the expression of more than 100 genes in *Streptococcus* and induce oxidative stress [67]. Similarly, although thiadiazole ring compounds have a broad spectrum of pharmacological activities including as anti-inflammatory, antiviral, and antibacterial agents [68], it is not currently known how the 1,2,3-thiadiazole ring compounds (HSI#1, 5 and 10) (Figure 6A) might affect protein secretion. Interestingly, thiouracil derivatives containing a triazolo-thiadiazole moiety have been developed and proposed to act as SecA inhibitors [22,64,69].

In summary, these results validated our new broad HTS approach by yielding starting molecules for potential new antibacterial development. A future focus of screening efforts to different exported reporter enzymes with different degrees of essentiality and topologies is expected to expand the gamut of promising compounds returned by this approach.

## Figures and Tables

**Table 1:** Properties of PhoA secretion inhibitors returned from the HTS.

Parent and Daughter Molecules	Secretion Inhibitor	Inhibition of PhoA Secretion IC_50_ [µM]			Bacterial Viability IC_50_ [µM]				Toxicity of Mammalian Cells (HEK293T) LD_50_ [µM]	Aqueous Solubility	
		HTS	Lab-Scale	Lab-Scale (Δ*tolC* strain)	*E. coli*			*B. subtilis* ATCC6633		µM	µg/mL
					BL21	MC4100	BW25113				
	CC#01 *	237	NA		4	4		>100	nt	nt	nt
	CC#02 **	24.5	NM		NM	NM		18.8	60.2	16.7	4.1
**Structure family 1**											
HTS hit	HSI#03	5.1	44.1	21.0	NM	40–100	NM	NM	60.2	150	40.0
**Structure family 2**											
HTS hit	HSI#07	19.0	26.4	19.9	NM	NM	NM	NM	60.2	150	32.4
Analog	HSI#12	11.5	11.2	26.0	60	NM	39.7	NM	60.2	50.0	11.8
Analog	HSI#14	13.4	14.6	11.5	>100	NM	31.3	NM	60.2	150.0	33.5
**Structure family 3**											
HTS hit	HSI#09	8.9	9.6	0.91	40–100	40–100	5.4	22.5	20.1	16.7	5.3
Analog	HSI#06	5.8	5.3	0.33	6.1	8.7	26.4	2.4	20.1	16.7	5.7
**Structure family 4**											
HTS hit	HSI#01	11.9	12.5	1.4	NM	NM	NM	37.6	6.7	50	12.5
Analog	HSI#05	32.0	7	0.94	NM	NM	NM	27.8	6.7	16.7	4.8
Analog	HSI#10	20.0	19.6	0.99	NM	NM	NM	26.0	6.7	16.7	4.4
**Structure family 5**											
HTS hit	HSI#11	3.0	NM	-	NM	NM	NM	NM	60.2	150.0	23.4
**Structure family 6**											
HTS hit	HSI#13	14.3	NM	-	NM	NM	NM	NM	60.2	150.0	57.7
Analog	HSI#08	17.0	NM	32.6	NM	NM	NM	NM	60.2	150.0	54.4
**Structure family 7**											
HTS hit	HSI#04	21.6	>50	7.8	NM	NM	NM	NM	60.2	150.0	37.6
**Structure family 8**											
HTS hit	HSI#02	56.7	NM	14.9	NM	NM	NM	NM	20.1	16.7	5.2

CC: Control compound. *: NaN_3_; concentration in (mM); value indicates MIC not IC_50_. **: Compound is PubChem ID 11528894, proposed as a low micromolar inhibitor of *E. coli* [48]. nt: Not tested. NA: Not applicable. NM: Non-measurable.

**Table 2:** Priority pathogens list for R&D of new antibiotics. Adjusted from the WHO 2018 recommendation list [41].

Pathogen List	Pathogen Used in this Study	Bacterial Viability (IC_50,_ µM)					
		CC#02 **	HSI#01	HSI#05	HSI#10	HSI#09	HSI#06
**Priority 1: Critical** Multidrug-resistant and extensively-resistant *Mycobacterium tuberculosis*							
*Mycobacterium tuberculosis*	*Mycobacterium abscessus* ATCC19977	NM	33.8	17.9	21.8	43.5	1.1
*Pseudomonas aeruginosa, carbapenem-resistant*							
*Pseudomonas aeruginosa*	*Pseudomonas aeruginosa* 3/88	NM	NM	NM	NM	NM	6.3
*Enterobacteriaceae, carbapenem-resistant, 3rd generation cephalosporin-resistant*							
*Klebsiella pneumoniae*	*Klebsiella pneumoniae* ATCC 27799	NM	80-100	NM	NM	80-100	8.7
Entheropathogenic *E. coli*	Entheropathogenic *E. coli* O127:H6 strain E2348/69	NM	NM	NM	NM	NM	>90
*Enterobacter cloacae*	*Enterobacter cloacae*	NM	NM	NM	NM	50	7.8
*Proteus vulgaris*	*Proteus vulgaris*	NM	80-100	NM	NM	34	2.9
*Providencia stuartii*	*Providencia stuartii*	NM	NM	NM	NM	NM	NM
*Morganella morganii*	*Morganella morganii*	NM	NM	NM	NM	70	6.4
*Serratia marcescens*	*Serratia marcescens*	NM	NM	NM	NM	NM	22.7
**Priority 2: High**							
*Enterococcus faecium*	*Enterococcus faecium* ATCC 804B	28.0	34.5	9.4	23.5	6.9	0.9
*Staphylococcus aureus*	*Staphylococcus aureus* ATC6538P	13.8	14.8	4.1	7.8	6.0	1.0
*Campylobacter* spp	*Campylobacter jejuni*	NM	NM	NM	NM	NM	NM
*Salmonella* spp	*Salmonella typhimurium*	NM	NM	NM	NM	80-100	12.4
**Priority 3: Medium**							
*Streptococcus pneumoniae*	*Streptococcus pneumoniae*	113.3	77.8	29.7	60.9	12.6	0.4
*Haemophilus influenzae*	*Haemophilus influenzae*	NM	NM	NM	NM	NM	NM
*Shigella* spp	*Shigella sonnei*	NM	NM	NM	NM	50	6.2

**: This compound is PubChem ID 11528894; proposed as a low micromolar inhibitor of *E. coli* secretion [48]. NM: Non-measurable.

## Data Availability

Not applicable.

## Supplementary Materials

The following are available online at https://www.mdpi.com/2076-2607/9/3/592/s1, Figure S1: Intracellular production of PhoA and effect of HSI#6 on growth of *E. coli* BW25113, Figure S2: Antibacterial activity of secretion inhibitors toward Gram-negative bacteria, Figure S3: Antibacterial activity of secretion inhibitors toward two Gram-positive bacterial species, Figure S4: Antibacterial activity of vancomycin, penicillin G and ampicillin toward *S. aureus*, Figure S5: Effect of compounds on SecA-dependent basal ATPase activities in vitro, Figure S6: Effect of compounds on Sec-dependent translocation ATPase in vitro, Table S1: Bacterial strains used in this study, supplementary materials and methods.
