# Supplementary material for: Effective Small Molecule Antibacterials from a Novel Anti-Protein Secretion Screen
Source: Microorganisms. 2021 Mar 13;9(3):592. doi: 10.3390/microorganisms9030592 (PMC8000395; doi:10.3390/microorganisms9030592)
Supplement: Supplementary file 1 [file microorganisms-09-00592-s001.pdf]

Supplementary materials

Effective small-molecule antibacterials from a novel anti-protein secretion screen

Mohamed Belal Hamed[1], Ewa Burchacka[2], Liselotte Angus[3], Arnaud Marchand[4], Jozefien De Geyter[5], Maria S. Loos[6], Jozef Anné[7], Hugo Klaassen[8], Patrick Chaltin[9], Spyridoula Karamanou[10] and Anastassios Economou[11*]

[1]Laboratory of Molecular Bacteriology, Rega Institute for Medical Research, KU Leuven, Belgium; Molecular Biology Department, National Research Centre, Dokii, Cairo, Egypt; mohamed.soliman@kuleuven.be

[2]Laboratory of Molecular Bacteriology, Rega Institute for Medical Research, KU Leuven, Belgium; Department of Microbiology and Medicinal Chemistry, Wroclaw University of Science and Technology, Wroclaw, Poland; ewa.burchacka@pwr.edu.pl

[3]Cistim Leuven vzw, Bioincubator 2, Gaston Geenslaan 2, 3001 Leuven, Belgium; liselotte.angus@gmail.com

[4]Cistim Leuven vzw, Bioincubator 2, Gaston Geenslaan 2, 3001 Leuven, Belgium; arnaud.marchand@cistim.be

[5]Laboratory of Molecular Bacteriology, Rega Institute for Medical Research, KU Leuven, Belgium; jozefien.degeyter@kuleuven.be

[6]Laboratory of Molecular Bacteriology, Rega Institute for Medical Research, KU Leuven, Belgium; maria.loos@kuleuven.be

[7]Laboratory of Molecular Bacteriology, Rega Institute for Medical Research, KU Leuven, Belgium; jozef.anne@kuleuven.be

[8]Cistim Leuven vzw, Bioincubator 2, Gaston Geenslaan 2, 3001 Leuven, Belgium; hugo.klaassen@cistim.be

[9]Cistim Leuven vzw, Bioincubator 2, Gaston Geenslaan 2, 3001 Leuven, Belgium; Center for Drug Design and Discovery (CD3), KU Leuven R&D, Leuven, Belgium; patrick.chaltin@kuleuven.be

[10]Laboratory of Molecular Bacteriology, Rega Institute for Medical Research, KU Leuven, Belgium; lily.karamanou@kuleuven.be

[11]Laboratory of Molecular Bacteriology, Rega Institute for Medical Research, KU Leuven, Belgium; tassos.economou@kuleuven.be

* **Correspondence:**Corresponding Author: tassos.economou@kuleuven.be

Table of contents

Abbreviations
Supplemental figures:
Figure S1 Intracellular production of PhoA and effect of HSI#6 on growth of *E. coli* BW25113.
Figure S2 Antibacterial activity of secretion inhibitors toward Gram-negative bacteria.
Figure S3 Antibacterial activity of secretion inhibitors toward two Gram-positive bacterial species.
Figure S4 Antibacterial activity of vancomycin, penicillin G and ampicillin toward *S. aureus*
Figure S5 Effect of compounds on SecA-dependent basal ATPase activities *in vitro.*
Figure S6 Effect of compounds on Sec-dependent translocation ATPase *in vitro*.

Supplemental tables:
Table S1 Bacterial strains used in this study

Supplementary Materials and methods:
Lab-scale assay of *in vivo* secretion of alkaline phosphatase
ATPase assay

References

Abbreviations:
ATPase – Adenosine triphosphatase
PhoA – Alkaline phosphatase
E. coli – Escherichia coli
EPEC – Entheropathogenic *E. Coli*
S. aureus – Staphylococcus aureus
B. subtilis – Bacillus subtilis
DMSO – dimethyl sulfoxide
IMVs – Inner Membrane Vesicles
ATP – Adenosine triphosphate
Tris – Tris(hydroxymethyl)aminomethane
DTT – 1,4-Dithiothreitol
BSA – Bovine serum albumin
Pi – Inorganic phosphate

Supplemental figures:

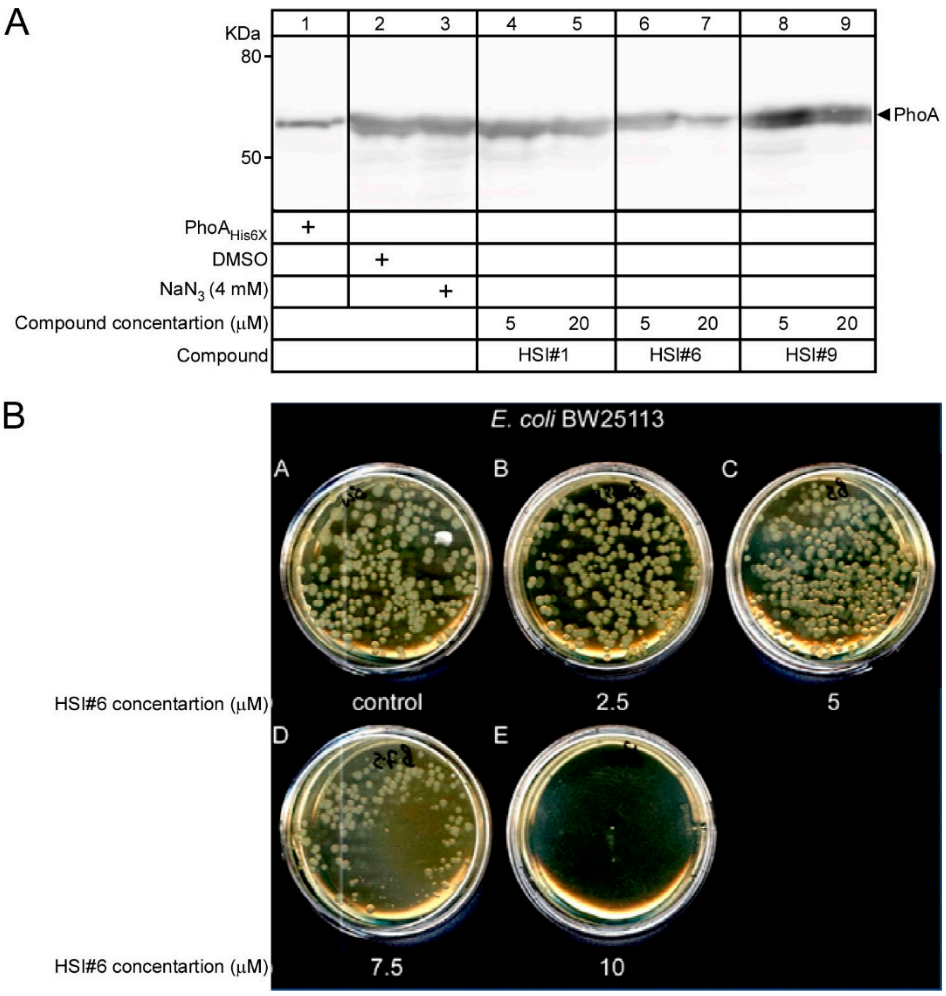

Figure S1. Intracellular production of PhoA and effect of HSI#6 on growth of *E. coli* BW25113.

A. *E. coli* BW25113 was grown in LB medium to OD_{600} 0.2 and incubated with either DMSO (0.67% *v/v*), NaN_3 (4 mM), compound (5 and 20 μM) and arabinose (1.33 μM) (see supplementary materials and methods). The cells were collected by centrifugation (4000 rpm, 4°C, 6 min.) and the pellet was washed twice with 50 mM Tris pH 8. Polypeptides were analyzed on 12% *w/v* acrylamide SDS-PAGE, followed by immunostaining with α-PhoA. PhoA is indicated.

B. Effect of HSI#6 on the growth of *E. coli* BW25113 on solid medium.

Growth of BW25113 was monitored on LB agar plates containing the indicated concentrations of HSI#6 dissolved in DMSO (1% v/v) or DMSO alone as a control. Plates were incubated for 24 h at 37°C.

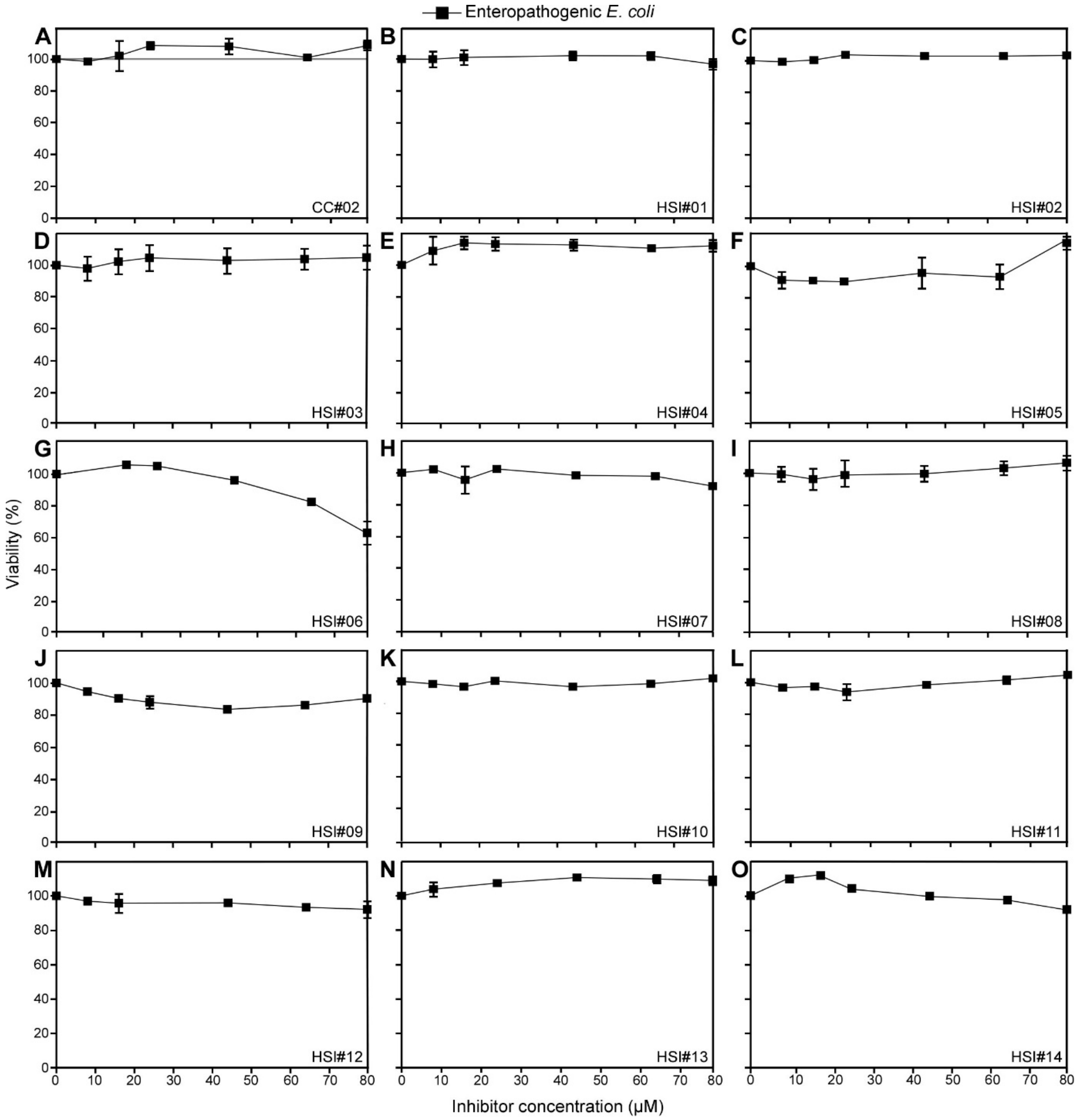

Figure S2. Antibacterial activity of secretion inhibitors **A-O** toward Gram-negative bacteria (related to Figure 2).

The measurements of inhibition of viability of EPEC were performed in duplicate. The results are presented as the mean ± SD, *n*=3. The growth of the bacteria (OD_{600} normalized to the 2.5% (v/v) DMSO) was plotted against the inhibitor concentration. The gray line in the left upper panel is shown as indicative for the growth in the presence of DMSO and the absence of inhibitor as a control.

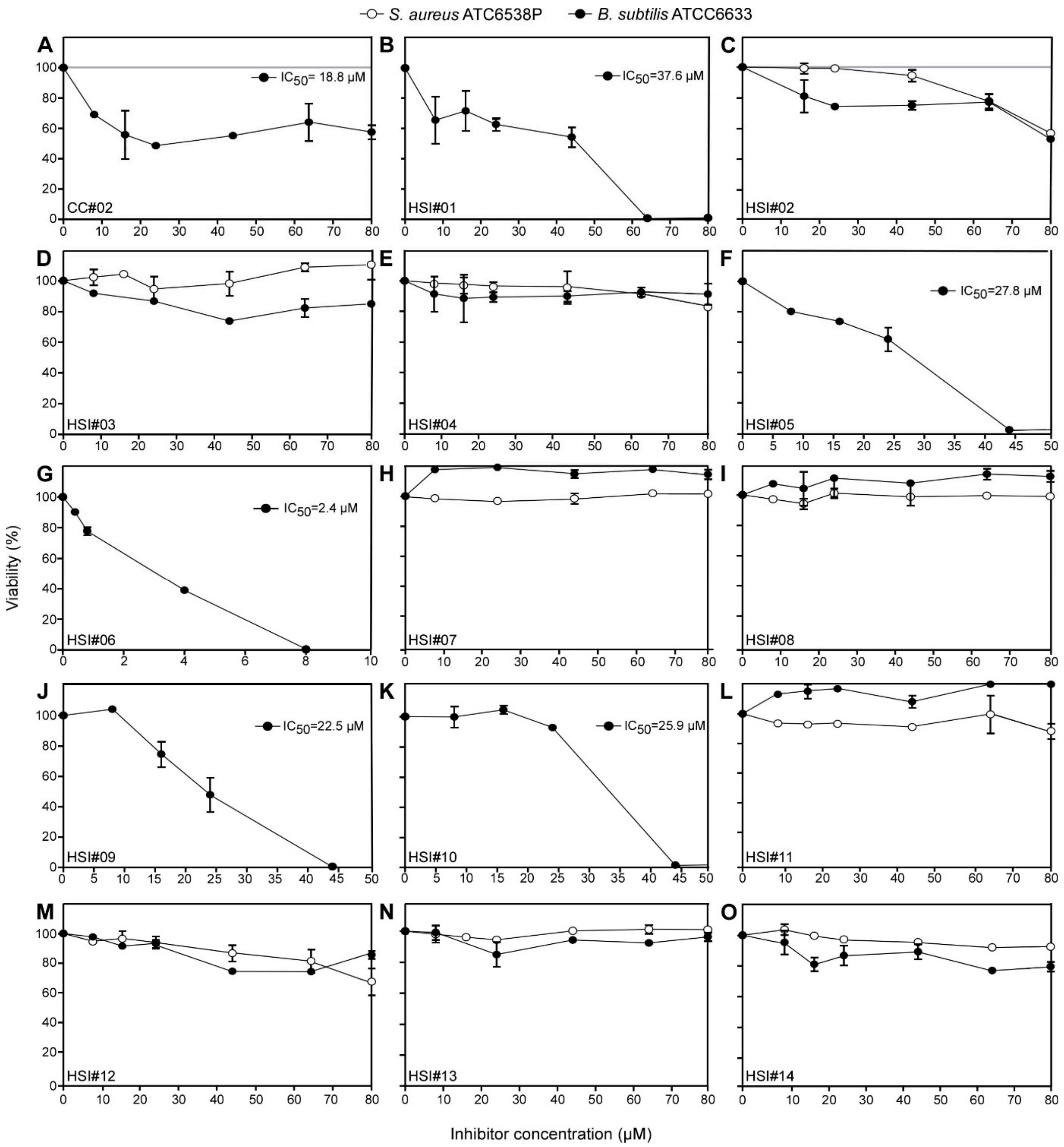

Figure S3. Antibacterial activity of secretion inhibitors **A**-**O** toward two Gram-positive bacterial species (related to Figure 3)

The measurements of inhibition on viability of *S. aureus* and *B. subtilis* were performed in duplicate. The results are presented as the mean ± SD, *n*=3. The growth of the bacteria (OD_{600} normalized to the 2.5% (v/v) DMSO) was plotted against the inhibitor concentration. The grey line in the left upper panel is shown as indicative for the growth in the presence of DMSO and the absence of inhibitor as a control.

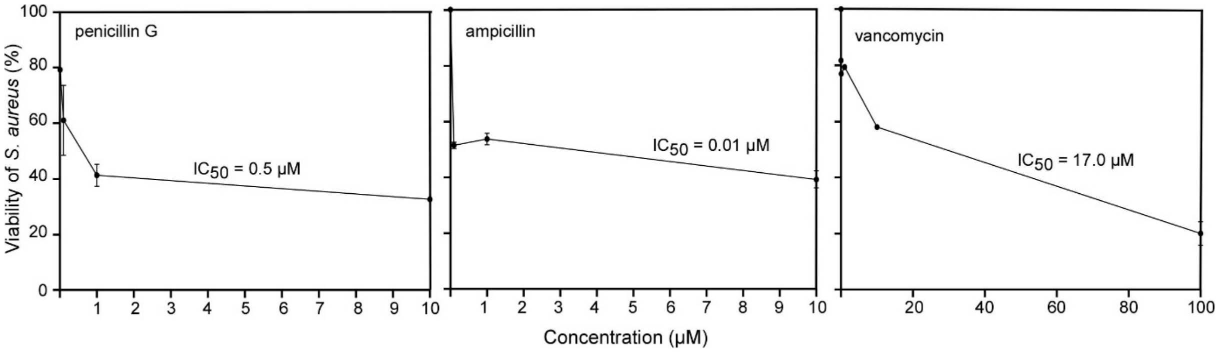

Figure S4. Antibacterial activity of vancomycin, penicillin G and ampicillin toward *S. aureus*

The measurements of inhibition on viability of *S. aureus* was performed in duplicate. The results are presented as the mean ± SD, *n*=3. The growth of the bacteria (OD_{600} nm normalized to the 2.5% (v/v) DMSO) was plotted against the concentration of the indicated antibiotic.

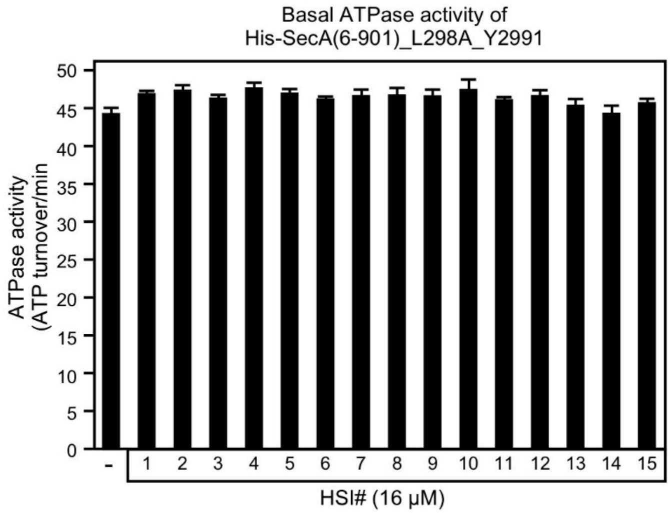

Figure S5. Effect of compounds on SecA-dependent basal ATPase activities *in vitro* (related to Figure 2).

The SecA basal ATPase assay was used to test whether the compounds have a direct inhibitory effect on SecA catalytic activity. The basal ATPase activity of His-SecA(6-901)_L298A_Y299A (abbreviated $SecA^{LY}$) was monitored in the absence or presence (16 µM) of compound. The $SecA^{LY}$ mutant version of SecA has an elevated Basal ATPase activity and allows to more easily detect changes in the Basal ATPase activity. None of the 14 compounds at the concentration used had any effect on the ATP turnover of $SecA^{LY}$. Hence, the antimicrobial effect and inhibitory effect on PhoA secretion of some compounds is not due to SecA inhibition.

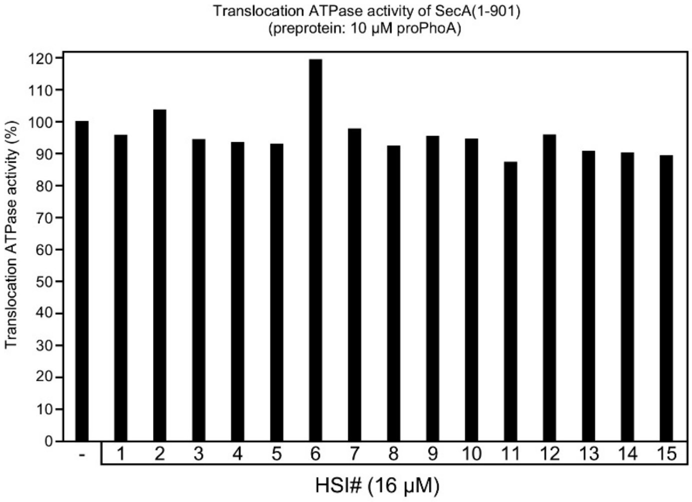

Figure S6. Effect of compounds on Sec-dependent translocation ATPase *in vitro* (related to Figure 2).

The effect of the compounds was tested on the Translocation ATPase of SecA (1-901) (abbreviated SecA). The amount of inorganic phosphate (Pi) released in the Translocation ATPase activity of SecA in presence of IMVs and proPhoA^{cys-} (as a preprotein) and 16 μM of each compound (See supplementary materials and methods below). The Translocation ATPase activity of SecA in the absence of compound and presence of DMSO was set as 100%. None of the 14 compounds (at 16 μM) had any effect on the Translocation ATP turnover of SecA. Hence, the antimicrobial effect and inhibitory effect on PhoA secretion of some compounds is not due to SecA inhibition.

Supplemental tables

Table S1 Bacterial strains used in this study.

Strains	Reference or Source
E. coli BL21	LMB stock collection
E.coli MC4100	LMB stock collection
Entheropathogenic *E. coli* O127:H6 strain E2348/69	G.Frankel (Imperial College, UK)
S. aureus ATC6538P	LMB stock collection
B.subtilis ATCC6633	LMB stock collection
Mycobacterium abscessus ATCC19977	Laboratory of clincal bacteriology and mycology (KU Leuven
Pseudomonas aeruginosa 3/88	LMB stock collection
Klebsiella pneumoniae ATCC 27799	LMB stock collection
Enterobacter cloacae	Laboratory medicine, KU Leuven
Proteus vulgaris	LMB stock collection
Providencia stuartii	Laboratory medicine, KU Leuven
Morganella morganii	Laboratory medicine, KU Leuven
Serratia marcescens	LMB stock collection
Enterococcus faecium ATCC 804B	LMB stock collection
Campylobacter jejuni	Laboratory medicine, KU Leuven
Salmonella typhimurium	LMB stock collection
Streptococcus pneumoniae	Laboratory medicine, KU Leuven
Haemophilus influenzae	Laboratory medicine, KU Leuven
Shigella sonnei	LMB stock collection

Lab-scale assay *of in vivo* secretion of alkaline phosphatase

E. coli MC4100 cells were transformed with a pIMBB1081 plasmid containing the pro(KL)PhoA-His gene and a preculture from a single colony (5 ml LB; 10 μg/ml gentamycin) was grown overnight at 30°C. A fresh 50 ml LB-gentamycin culture was inoculated (1:50) with the preculture and grown at 30°C to OD_{600} 0.2. The culture was split onto a microplate (200 μl per well) and either dH_2O; NaN_3 (4 mM) and arabinose (1.33 μM); arabinose (1.33 μM); compound (3-100 μM) and arabinose (1.33 μM) were added. All conditions had 0.67% DMSO. The microplate was incubated for 3 hours (30°C; 180 rpm). The samples of each well were centrifuged (4000 rpm, 4°C, 6 min.) and the pellet was resuspended in 200 μl 50 mM Tris pH 8. The samples were diluted (1:17) and 250 μl was used to measure OD_{600}. PNPP (10 mM) was added to another 500 μl and these samples were incubated for 5 minutes at 37°C. Then they were transferred on ice and K_2HPO_4 (160 mM) and Triton (0.17%) were added to block the alkaline phosphatase and lyse the cells respectively. 250 μl of the sample, after being centrifuged at 15 000 g (5 min; 4°C), was used to measure OD_{420}. The PhoA activity was calculated using the formula: $(1000 \times OD_{420} \times \text{dilution factor}) / (\text{incubation time} \times OD_{600})$.

ATPase assay

The ATPase assay measures the activity of SecA by monitoring the amount of inorganic phosphate (Pi) released. Basal (SecA alone), Membrane (SecA in presence of Inner Membrane Vesicles (IMVs)) and Translocation (SecA in presence of IMVs and preprotein) ATPase were determined. The ATPase buffer contains 0.4 μM SecA, 1 mM ATP, 50 mM Tris pH 8, 50 mM KCl, 5 mM $MgCl_2$, 1 mM DTT and 0.4 mg/ml BSA (Basal). To obtain the Membrane ATPase, diluted IMVs overexpressing SecYEG were added. To obtain the Translocation ATPase, $\text{proPhoA}^{\text{cys-}}$ (in 6M urea buffer; 10 μM) was added (the final concentration of urea in the ATPase buffer cannot exceed 0.2 M final concentration). To test the effect of the compounds (stored in 100% DMSO), 16 μM of each compound was added in the Basal and Translocation ATPase reactions (all Basal, Membrane and Translocation reactions were adjusted to have 2% DMSO final concentration). For the Basal ATPase, a SecA mutant (His-SecA $(6\text{-}901)^{\text{L2981\_Y299A}}$) with elevated ATPase activity was used. For the Translocation ATPase, wild-type SecA(1-901) was used. The ATPase samples were incubated for 20 minutes (Basal and Membrane) or 10 minutes (Translocation) at 37°C and then immediately transferred on ice. The released Pi was detected spectrophotometrically (660 nm) using the malachite green reagent. The ATP turnover (K_{cat}) (mol Pi/mol SecA protomer/min) values for the Basal, Membrane and Translocation SecA ATPase activity were determined. The Basal ATPase is low (4 min^{-1}), IMVs stimulate ATPase activity (ratio Membrane ATPase/Basal ATPase is 1.2). When SecYEG:SecA molar ratio is equimolar, the Membrane ATPase of SecA is 1.2x over its Basal ATPase [1]. In presence of preprotein, the ATPase activity of SecA is stimulated significantly (ratio Translocation ATPase/Membrane ATPase = ± 10 when ratio Membrane ATPase/Basal ATPase is 1.2).

References

1. Gouridis, Giorgos, Spyridoula Karamanou, Marios Frantzeskos Sardis, Martin Alexander Schärer, Guido Capitani, and Anastassios Economou. "Quaternary Dynamics of the Seca Motor Drive Translocase Catalysis." *Molecular Cell* 52, no. 5 (2013): 655-66.
